# Progress Toward Poliomyelitis Eradication — Pakistan, January 2012–September 2013

**Published:** 2013-11-22

**Authors:** World Health Organization (WHO) Country Office, Islamabad, Pakistan, Polio Eradication Dept, WHO Eastern Mediterranean Regional Office, Cairo, Egypt, Polio Eradication Dept, WHO, Geneva, Switzerland, Div of Viral Diseases, National Center for Immunization and Respiratory Diseases, Global Immunization Div, Center for Global Health, CDC

Pakistan is one of three countries where transmission of indigenous wild poliovirus (WPV) has never been interrupted (1). This report describes polio eradication activities and progress in Pakistan during January 2012–September 2013 and updates previous reports (2,3). During 2012, 58 WPV cases were reported in selected areas, compared with 198 cases throughout the country in 2011; 52 WPV cases were reported during January–September 2013, compared with 54 cases during the same period in 2012. Of the 110 WPV cases reported since January 2012, 92 cases (84%) occurred in the conflict-affected Federally Administered Tribal Areas (FATA) and in security-compromised Khyber Pakhtunkhwa (KP) Province. WPV type 3 (WPV3) was isolated from only three persons with polio in a single district in 2012; the most recent case occurred in April 2012. During August 2012–September 2013, 52 circulating vaccine-derived poliovirus type 2 (cVDPV2) cases were detected, including 30 cases (58%) identified in FATA during January–September 2013. Approximately 350,000 children in certain districts of FATA have not received polio vaccine during supplementary immunization activities (SIAs)* conducted since mid-2012 because local authorities have banned polio vaccination. In some other areas of Pakistan, SIAs have been compromised by attacks targeting polio workers that started in mid-2012. Further efforts to reach children in conflict-affected and security-compromised areas, including vaccinating at transit points and conducting additional short-interval-additional-dose (SIAD)† SIAs as areas become accessible, will be necessary to prevent reintroduction of WPV into other areas of Pakistan and other parts of the world.

## Immunization Activities

Estimated national routine vaccination coverage among infants aged <1 year with 3 doses of oral polio vaccine (OPV3) was 89% in 2012, unchanged from 2011 (4). However, based on parental recall and immunization cards, routine OPV3 coverage among children aged 6–23 months with nonpolio acute flaccid paralysis (NPAFP)§ was 65% nationally, with a large range among provinces and territories: 28% in Balochistan, 38% in FATA, 54% in Sindh, 57% in KP, 78% in Punjab, and 89% in Azad Jammu and Kashmir, Gilgit-Baltistan, and Islamabad Capitol Territory combined.

**FIGURE 1:**
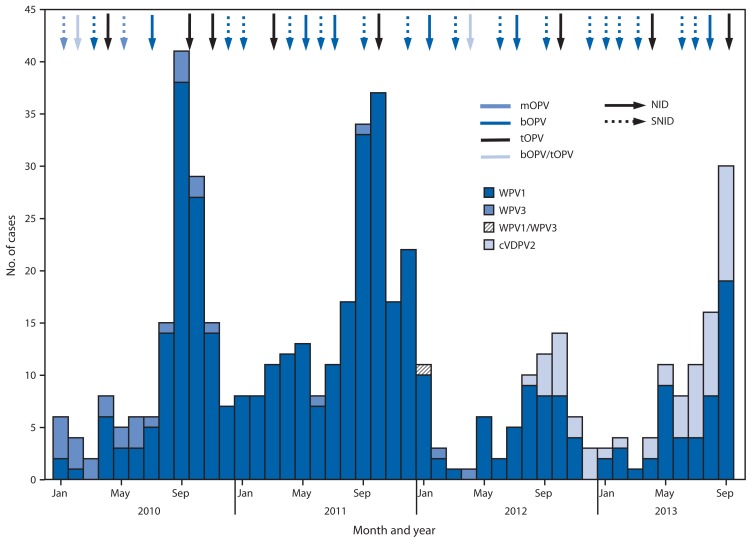
Number of cases of wild poliovirus types 1 (WPV1), 3 (WPV3), 1 and 3 (WPV1/WPV3), and circulating vaccine-derived poliovirus type 2 (cVDPV2), type of supplementary immunization activity conducted, and type of vaccine used, by month — Pakistan, 2010–2013* **Abbreviations:** NID = national immunization days; SNID = subnational immunization days; mOPV = monovalent oral poliovirus vaccine; bOPV = bivalent oral poliovirus vaccine; tOPV = trivalent oral poliovirus vaccine. * Data as of November 4, 2013.

During January 2012–September 2013, seven national and nine subnational SIAs targeting children aged <5 years were conducted. Three national SIAs used trivalent OPV, three national and all subnational SIAs used bivalent OPV types 1 and 3 (bOPV), and one national SIA used both vaccines (in different areas) (Figure 1). Eleven SIAD SIAs and several smaller mop-up campaigns using bOPV or monovalent OPV type 1 were conducted, targeting areas with recent confirmed polio cases or districts with children at high risk for polio.

During January–July 2012, an estimated 15% of children targeted during SIAs (nearly 170,000 children) in FATA were not accessible because of security limitations for vaccination teams.¶ From July 2012 to September 2013, the estimated percentage of targeted children living in SIA-inaccessible areas of FATA increased to 33%–35% (approximately 377,000 to 400,000 children) because of security limitations, bans on polio vaccination by local authorities, or both. In additional areas of FATA and in Sindh and KP provinces, targeted attacks against polio workers during SIAs from July 2012 to September 2013 increased costs, limited full implementation, and prevented monitors and supervisors from assessing the quality and coverage of SIAs.

Nationally, using acute flaccid paralysis (AFP) surveillance data to provide a proxy measure of OPV coverage, 95% and 92% of children aged 6–23 months with NPAFP were reported to have received ≥4 OPV doses through routine vaccination or SIAs in 2012 and 2013, respectively. Only 2% and 5% of children with NPAFP had never received a dose of OPV (“zero-dose children”) in 2012 and 2013. However, the percentage of children with NPAFP who received ≥4 OPV doses was >90% in Azad Jammu and Kashmir, Gilgit-Baltistan, Islamabad Capitol Territory, KP, Punjab, and Sindh during 2012 and 2013; it was only 78% in Balochistan during both years; and in FATA, it declined from 71% in 2012 to 35% in 2013. The proportion of zero-dose children among those with NPAFP increased in FATA, from 17% in 2012 to 52% in 2013, but remained unchanged in Balochistan (10%), KP (<5%), and elsewhere (<1%).

## Poliovirus Surveillance

Standard indicators are used to monitor AFP surveillance performance globally (5).** In 2012, the annual national NPAFP rate in Pakistan (per 100,000 population aged <15 years) was 6.3 (range among the six provinces/territories: 2.4–9.1). The percentage of AFP cases for which adequate specimens were collected was 89% (range: 73%–92%) (Table).

During 2012–2013, to supplement AFP surveillance in Pakistan, environmental surveillance with monthly testing of sewage samples for polioviruses was conducted in 23 sites in 11 cities in all major provinces of Pakistan. During 2011, WPV1 was detected from all sites, and 136 (65%) of 205 samples collected were positive. During 2012–2013, WPV1 was isolated in nearly all samples in Peshawar (KP) and Hyderabad (Sindh), but the frequency of isolation declined in other sites. In 2012, 87 (36%) of 239 samples were WPV1-positive; during January–September 2013, 40 (16%) of 247 samples were WPV1-positive, compared with 74 (40%) of 187 samples collected during the same period in 2012.

## WPV and Vaccine-Derived Poliovirus (VDPV) Epidemiology

**FIGURE 2:**
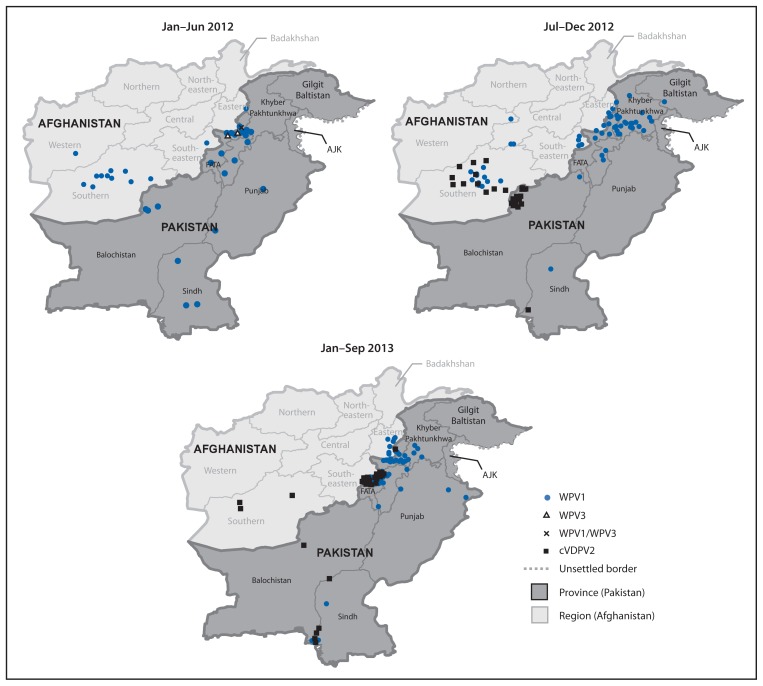
Cases of wild poliovirus types 1 (WPV1), 3 (WPV3), 1 and 3 (WPV1/WPV3), and circulating vaccine-derived poliovirus type 2 (cVDPV2) — Pakistan, January 2012–September 2013*^†^ **Abbreviations:** FATA = Federally Administered Tribal Areas; AJK = Azad Jammu and Kashmir. * Data as of November 4, 2013. † Each dot represents one poliovirus case. Dots drawn at random within districts.

During 2012, 58 WPV cases (55 WPV1, two WPV3, and one WPV1/WPV3 coinfection) were reported, compared with 198 WPV cases (196 WPV1 and two WPV3) during 2011; 52 cases (all WPV1) were reported during January–September 2013, compared with 54 cases for the same period in 2012 (Table, Figures 1 and 2). Of 110 WPV cases reported during January 2012–September 2013, 96 (87%) cases were among children aged <36 months; 45 (41%) were reported to have received no OPV doses, 16 (15%) received 1–3 OPV doses, and 49 (45%) received ≥4 OPV doses.

WPV cases were reported in 27 (17%) of 157 districts during 2012, compared with 60 (38%) districts during 2011, and from 16 (10%) districts during January–September 2013. During 2012, 27 (47%) of 58 WPV cases were from KP, 20 (34%) from FATA, and eight (14%) from Balochistan and Sindh combined (Table, Figure 2). During January–September 2013, 36 (69%) of 52 cases were from FATA, nine (17%) from KP, four (8%) from Sindh, and none from Balochistan.

WPV genomic sequencing identified six genetic clusters†† of WPV1 during 2012 and four clusters during January–September 2013. During 2013, some clusters have only been detected in sewage samples but not in specimens from AFP cases. During 2012–2013, only three WPV3 cases were reported; all were from the same district in FATA (Khyber), and the WPV3 isolates belonged to a single genetic cluster. The date of onset for the most recent WPV3 case was April 2012. The latest WPV3 isolated from a sewage sample was collected in Karachi in October 2010.

The first circulating VDPV-associated polio case ever reported in Pakistan was detected in Killa Abdullah District, Balochistan, with onset on August 30, 2012 (6). Genomic sequencing suggested that circulation was undetected for nearly 2 years since emergence. During August 2012–September 2013, 52 cVDPV2 cases were reported: 17 in Balochistan, 30 in FATA (primarily in areas where vaccination teams do not have access), and five in Sindh (Table, Figures 1 and 2). Of the 52 cases reported, 47 (90%) were among children aged <36 months; 26 (50%) had received zero OPV doses (either routine or SIA), and 17 (33%) had received ≥4 OPV doses.

### Editorial Note

During 2012, WPV cases declined 70% compared with 2011 (58 cases versus 198 cases) and were more geographically restricted. In 2013 to date, a similar number of WPV cases were reported compared with a similar period in 2012, with further geographic restriction. In 2013, AFP and environmental surveillance suggest that WPV1 transmission generally has been restricted to high-risk areas of FATA and KP, and WPV1 circulation apparently has been interrupted in the Quetta block, Balochistan, one of the historical reservoir areas. WPV3 has not been detected in any stool or sewage sample in Pakistan for >1 year. During 2012, however, cVDPV2 emerged in the Quetta block because of long-standing, low routine vaccination coverage and poor-quality SIAs. More importantly, during 2013, WPV1 transmission in FATA has intensified, and cVDPV2 originating from Quetta block has quickly spread in certain areas of FATA where conflict and local bans on polio vaccination have prevented access by vaccination teams for >1 year.

What is already known on this topic?

Pakistan is one of the three remaining countries (including Afghanistan and Nigeria) where indigenous wild poliovirus (WPV) transmission has never been interrupted. Pakistan has been the source for imported WPV outbreaks in Afghanistan, China, and Syria and for WPV circulation in Egypt, Israel, the West Bank, and Gaza.

What is added by this report?

Compared with the same period in 2010–2011, WPV type 1 transmission decreased in magnitude and geographic spread, and WPV type 3 has not been detected for >1 year. However, bans on polio vaccination and attacks targeting polio workers in certain areas have resulted in intense WPV type 1 transmission and rapid spread of circulating vaccine-derived poliovirus type 2, especially in the Federally Administered Tribal Areas (FATA).

What are the implications for public health practice?

Improvements in polio program performance and the decreased extent of WPV transmission in Pakistan suggest that the successful eradication of polio is achievable. The intense transmission of WPV type 1 and circulating vaccine-derived poliovirus type 2 in FATA, with transmission within and outside Pakistan, demonstrates the ongoing threat to achievement of polio eradication. Efforts by humanitarian, religious, and governmental bodies to improve community acceptance of vaccination and to reach children in conflict-affected and security-compromised areas of Pakistan will be necessary to achieve polio eradication in Pakistan and globally.

During 2010 and 2011, WPV cases increased substantially in number and dispersion, spreading throughout the country (2,3). This surge in WPV cases was attributed primarily to population displacement after severe flooding in 2010, low routine OPV3 vaccination coverage, and the low quality of SIAs because of insufficient political involvement at the federal, provincial, and district levels. The quality of SIAs improved in 2012, after implementation of management and accountability strategies included in the 2012 Enhanced National Emergency Action Plan (7). However, since July 2012, targeted attacks, resulting in the death of 22 polio workers and four police officers and the injury of many others, have seriously compromised implementation of SIAs in many areas of FATA, KP, and Karachi. SIAs were resumed in some areas of FATA, KP, and Karachi after initial suspension after the attacks against polio workers. However, the quality of vaccination activities in these areas likely has been reduced because of strategies implemented to minimize the risk for attacks, such as conducting SIAs without advance notice, reducing or suspending house-to-house visits in some locales, and having police escorts for vaccination teams. Furthermore, cancellation of post-SIA surveys prevented assessments of SIA quality and management of vaccination team performance problems. In addition, in the North and South Waziristan agencies of FATA, bans by local authorities have prohibited polio vaccination for approximately 350,000 children since June 2012.

Major improvements in polio program performance and the decreased extent of transmission of WPV in Pakistan suggest that the successful eradication of polio is achievable. However, the high proportion of children infected with WPV or with NPAFP who are underimmunized and the simultaneous WPV1 and cVDPV2 outbreaks in FATA during 2013 highlight the serious consequences to population immunity that have resulted from conflict and insecurity. WPV1 from inaccessible areas of FATA has spread to other areas in Pakistan and to other countries. All WPV1 cases in Afghanistan in 2013 have occurred in the Eastern Region adjoining FATA and were caused by WPV1 originating in FATA. Recent WPV1 transmission in Egypt, Israel, the West Bank and Gaza, and Syria (8) can be linked to WPV1 originating in Pakistan. This situation puts recent achievements in Pakistan at risk for reversal and puts achievement of the objective of global polio eradication in peril. Enhanced efforts by humanitarian, religious, and governmental bodies to improve community acceptance of vaccination and reach children in conflict-affected and security-compromised areas of Pakistan will be necessary to interrupt all poliovirus transmission in Pakistan.

## Figures and Tables

**TABLE:** Acute flaccid paralysis (AFP) surveillance indicators and reported cases of wild poliovirus (WPV) and circulating vaccine-derived poliovirus type 2 (cVDPV2), by province, period, and poliovirus type — Pakistan, January 2012–September 2013*

				Reported WPV cases						Reported cVDPV2 cases	
			
	AFP surveillance indicators (2012)			Period			Type			Period	
				
Country/Area	No. of AFP cases	Nonpolio AFP rate†	% with adequate specimens§	Jan–Jun 2012	Jul–Dec 2012	Jan–Sep 2013	WPV1	WPV3	WPV1/WPV3¶	Jul–Dec 2012	Jan–Sep 2013
**Pakistan**	**5,037**	**6.3**	**89**	**24**	**34**	**52**	**107**	**2**	**1**	**16**	**36**
AJK, GB, ICT	74	2.4	92	0	1	0	1	0	0	0	0
KP	990	9.1	85	5	22	9	36	0	0	0	0
FATA	149	7.9	73	11	9	36	53	2	1	0	30
Punjab	2,407	5.8	91	2	0	3	5	0	0	0	0
Balochistan	205	5.0	83	3	1	0	4	0	0	15	2
Sindh	1,212	6.8	90	3	1	4	8	0	0	1	4

**Abbreviations:** AJK = Azad Jammu and Kashmir; GB = Gilgit-Baltistan; ICT = Islamabad Capital Territory; KP = Khyber Pakhtunkhwa (formerly Northwest Frontier Province); FATA = Federally Administered Tribal Areas.

* Data as of November 4, 2013.

† Per 100,000 children aged <15 years.

§ Two stool specimens collected ≥24 hours apart, both within 14 days of paralysis onset, and shipped on ice or frozen packs to a World Health Organization–accredited laboratory, arriving in good condition.

¶ One case had coinfection with WPV1 and WPV3.

## Footnotes

* Mass campaigns conducted for a brief period (days to weeks) in which 1 dose of oral poliovirus vaccine is administered to all children aged <5 years, regardless of vaccination history. Campaigns can be conducted nationally or in portions of the country.

† Short-interval-additional-dose campaigns are used during negotiated periods of nonviolence in otherwise inaccessible areas to administer a second dose of monovalent oral polio vaccine or bivalent oral polio vaccine within 1–2 weeks of the previous dose.

§ Vaccination histories of children aged 6–23 months with acute flaccid paralysis who do not test WPV-positive are used to estimate OPV coverage of the overall target population and to corroborate national reported routine vaccination coverage estimates.

¶ Areas considered too dangerous by the World Health Organization and the local government to conduct an SIA.

** The quality of AFP surveillance is monitored by performance indicators that include 1) detection rate of NPAFP cases and 2) the proportion of AFP cases with adequate stool specimens. World Health Organization (WHO) operational targets for countries with endemic poliovirus transmission are an NPAFP detection rate of at least two cases per 100,000 population aged <15 years and adequate stool specimen collection from >80% of AFP cases, in which two specimens are collected at least 24 hours apart, both within 14 days of paralysis onset, and shipped on ice or frozen packs to a WHO-accredited laboratory, arriving in good condition.

†† All WPVs isolated are sequenced across the interval encoding the major capsid protein (VP1) (approximately 900 nucleotides), and results are analyzed to monitor pathways of virus transmission. Isolates within a cluster share >95% VP1 nucleotide sequence identity.
